# Antiviral Lead Compounds from Marine Sponges

**DOI:** 10.3390/md8102619

**Published:** 2010-10-11

**Authors:** Sunil Sagar, Mandeep Kaur, Kenneth P. Minneman

**Affiliations:** Computational Bioscience Research Center, King Abdullah University of Science and Technology, Thuwal 23955-6900, Jeddah, Saudi Arabia; E-Mails: mandeep.kaur@kaust.edu.sa (M.K.); kenneth.minneman@kaust.edu.sa (K.P.M.)

**Keywords:** sponge, natural product, antiviral, metagenomics

## Abstract

Marine sponges are currently one of the richest sources of pharmacologically active compounds found in the marine environment. These bioactive molecules are often secondary metabolites, whose main function is to enable and/or modulate cellular communication and defense. They are usually produced by functional enzyme clusters in sponges and/or their associated symbiotic microorganisms. Natural product lead compounds from sponges have often been found to be promising pharmaceutical agents. Several of them have successfully been approved as antiviral agents for clinical use or have been advanced to the late stages of clinical trials. Most of these drugs are used for the treatment of human immunodeficiency virus (HIV) and herpes simplex virus (HSV). The most important antiviral lead of marine origin reported thus far is nucleoside Ara-A (vidarabine) isolated from sponge *Tethya crypta*. It inhibits viral DNA polymerase and DNA synthesis of herpes, vaccinica and varicella zoster viruses. However due to the discovery of new types of viruses and emergence of drug resistant strains, it is necessary to develop new antiviral lead compounds continuously. Several sponge derived antiviral lead compounds which are hopedto be developed as future drugs are discussed in this review. Supply problems are usually the major bottleneck to the development of these compounds as drugs during clinical trials. However advances in the field of metagenomics and high throughput microbial cultivation has raised the possibility that these techniques could lead to the cost-effective large scale production of such compounds. Perspectives on biotechnological methods with respect to marine drug development are also discussed.

## 1. Introduction

Marine sponges (phylum Porifera) are among the oldest multicellular invertebrate organisms [1] exhibiting a wide variety of colors and shapes. About 8,000 species of sponges, inhabiting different marine and freshwater ecosystems have been described to date [2]. Marine sponges are a rich source of potent natural products, some of which are considered as highly significant lead compounds for drug development. Most of these are secondary metabolites produced by the sponges [3] which may be produced to defend themselves against pathogenic bacteria, algae, fungi and other potential predators; a system they have developed during the process of evolution throughout thousands of years. More than 5,300 different natural compounds have been discovered from sponges and their associated microorganisms, and every year several hundred new compounds are being added [4].

Antiviral compounds are currently of particular interest since viral diseases (e.g., HIV, H1N1, HSV, *etc.*) have become major human health problems in recent decades. The ability of a virus to rapidly evolve and develop resistance to existing pharmaceuticals calls for continuing development of new antiviral drugs. Several lead antiviral compounds have been isolated from marine sponges, and there has been a consistent effort to identify new compounds.

The nucleosides spongothymidine and spongouridine were the first compounds isolated from a marine sponge (*Tethya crypta*) [5,6] which further led to the synthesis of Ara-C, an anticancer agent and Ara-A, the first antiviral drug. Ara-A inhibits viral DNA synthesis by conversion into adenine arabinoside triphosphate which inhibits viral DNA polymerase and DNA synthesis of herpes, vaccinica and varicella zoster viruses. It has been used clinically for treatment of herpes virus infection. Ara-A was the only sponge derived compound which was approved by the US FDA as an antiviral drug, although its marketing was later stopped as it was found to be less efficient and more toxic than the newer drug acyclovir (Zovirax) [7,8]. In addition to nucleosides, marine sponges are also the source of many alkaloids, sterols, terpenes, peptides, fatty acids, peroxides, *etc.* exhibiting the remarkable chemical diversity of compounds found in these organisms [9].

Several other sponge derived antiviral compounds are in preclinical/clinical trials for various diseases. However significant problems associated with these compounds have been a major limitation in the drug development and approval process. This is primarily due to the many technological challenges in detecting, isolating, characterizing, and scaling up production of bioactive compounds from marine sponges. To solve the critical supply problem, several efforts are being made in sponge farming, metagenomics and microbial cultivation, which are discussed below. Here we focus on existing or promising antiviral lead compounds from marine sponges which may have the potential to be future drugs.

## 2. Antiviral Lead Compounds

### 2.1. Nucleosides

#### 2.1.1. Vidarabine or Ara-A

**Figure 1:**
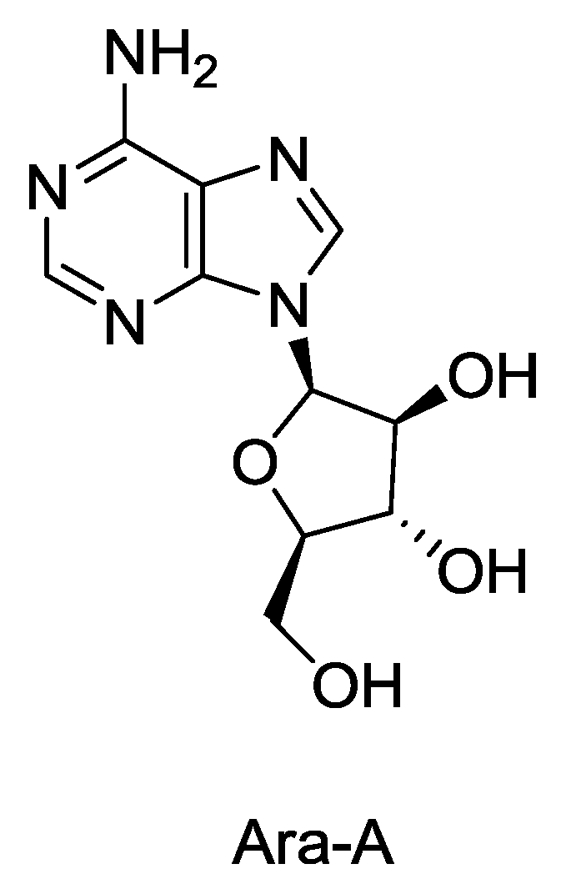
Structure of Ara-A.

In 1950, Bergmann *et al*. [6] isolated from the Caribbean sponge *Tethya crypta* (Tethylidae) the nucleosides spongothymidine and spongouridine, which contained an arabinose sugar rather than the more common ribose sugar found in these nucleosides. Vidarabine or Ara-A is a synthetic analogue of spongouridine with improved antiviral activity. The antiviral activity of adenine arabinoside (vidarabine, Ara-A, Figure 1) was first described by Privat de Garilhe and De Rudder in 1964 [10]. The work of Whitley in 1976 further confirmed the clinical usefulness of the compound in the treatment of herpes encephalitis and the other herpes infections that occasionally occur in newborns [11]. It was the first nucleoside antiviral to be licensed for the treatment of systematic herpes virus infection and one of the three marine-derived drugs currently approved by the FDA in the United States [12], however the marketing of the drug has been discontinued because the availability of newer and better antiviral agents on the market.

Despite its proven ability as a therapeutic agent which is active against a variety of viruses, vidarabine has some significant limitations. It is readily metabolized by adenosine deaminase (ADA) to arabinofuranosyl hypoxanthine (ara-H), which is 10-fold less potent [13,14] and has low lipophilicity and thus low intestinal membrane permeability. It is also poorly soluble in aqueous solutions and has low intramuscular absorption, requires large fluid volumes for intravenous administration, and must be given over prolonged periods (8 to 12 h) [15] to obtain therapeutic effects. Later acyclovir (Zovirax) was found to be a better drug than vidarabine for the treatment of herpes virus infections [7,8] however vidarabine was reported to be capable of inhibiting acyclovir-resistant HSV and VZV (varicella-zoster virus) [16,17]. Vidarabine is an inhibitor of viral DNA synthesis [18]. Adenine arabinoside (vidarabine) is converted into adenine arabinoside triphosphate (ara-ATP) *in vivo* [19] by kinases encoded by viruses, which in turn inhibit viral DNA polymerase and hence DNA synthesis of herpes, vaccinia and varicella zoster viruses [12,20]. Another study found that vidarabine is incorporated into RNA as well as DNA, leading to another possible mechanism of action of the drug. It was observed that vidarabine inhibited the initial RNA polyadenylation reaction catalyzed by chromatin-bound poly (A) polymerase [21]. It was also recently reported that vidarabine was 3–5 fold more active in plaque reduction assays against vaccinia and cowpox viruses than was cidofovir (Vistide) [22].

Stereocontrolled synthesis of vidarabine [23] and several analogues/derivatives with antiviral activity has been described [24–26]. Its biosynthesis from *Streptomyces antibioticus* has also been reported [27].

#### 2.1.2. Mycalamide A, Mycalamide B

**Figure 2:**
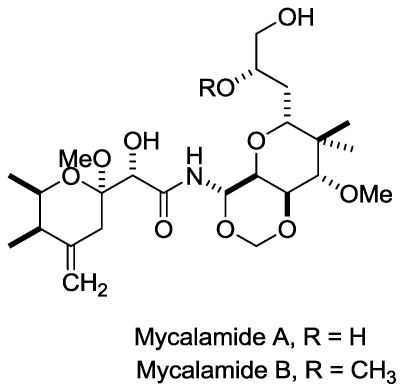
Structures of mycalamide A and B.

Perry *et al.* [28] first reported the isolation and *in vitro* antiviral activity of mycalamide A and mycalamide B (Figure 2) from a New Zealand sponge of the genus *Mycale* in 1988 and 1990, respectively. The crude extract containing 2% mycalamide A was found to be active against A59 corona virus. After treatment with crude extract at 0.1 mg/kg, mice infected with virus survived for 14 days, however the mice infected with virus died within eight days. Mycalamide A also inhibited the Herpes simplex type I and Polio type I viruses at a concentration of 5 ng/disc. Mycalamide B was found to be more potent than mycalamide A, which was active at a concentration of 1–2 ng/disc [29]. Examining the mechanisms involved in the actions of these compounds, Burres and Clement discovered the inhibition of protein synthesis and translation of RNA into protein in a cell-free lysate of rabbit reticulocytes [30]. A new study also described the binding of mycalamide A to the E site of the large ribosomal subunit of *Haloarcula marismortui* and inhibition of protein synthesis [31]. This property of protein synthesis inhibition may be attributed to their biological activity as antiviral agents.

Several studies regarding the total synthesis of mycalamides have been published [32–35]. Four analogues of mycalamide A have recently been reported [36] to bind the nucleoprotein (NP) of influenza virus and inhibit its multiplication. It has also shown experimentally that these compounds might bind to the N-terminal 13-amino acid region of NP which mediates the nuclear transport of NP and its binding to viral RNA, and hence may inhibit viral replication [36].

### 2.2. Sesquiterpene Hydroquinones

#### Avarol

**Figure 3:**
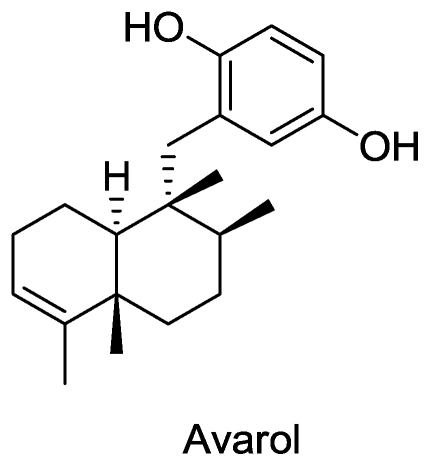
Structure of avarol.

Avarol, a sesquiterpenoid hydroquinone with a rearranged drimane skeleton, was first isolated from the marine sponge *Disidea avara* in 1974 [37]. The chemical structure of avarol (Figure 3) was established by standard analytical methods and chemical degradation [38] and by its stereocontrolled total synthesis [39].

The compound showed a dose-dependent inhibitory effect on the replication of the etiologic agent of acquired immune deficiency syndrome (AIDS) and human T-lymphotropic retrovirus (HTLV III)/lymphadenopathy-associated virus in human H9 cells *in vitro* at a concentration of 0.1 μg/mL [40]. The study suggested that the mechanism involved blocking the expression of the p24 and p17 gag proteins of HTLV-III in H9 cells after virus infection, and hence blocking viral replication. Studies dating back to 1988 showed that the antiviral effects of avarol were due to an increase in intracellular levels of superoxide radicals such as superoxide dismutases and of glutathione peroxidase [41]. The effects of avarol were further elucidated and it was found that it completely blocks the synthesis of glutamine transfer tRNA, which is crucial for synthesis of a viral protease required for viral proliferation [42–44]. Other important biological targets inhibited by avarol or its derivatives include reverse transcriptase [45] which plays a key role in early stages of viral infection, inhibition of cyclooxygenase and 5′-lipoxygenase, thus reducing the levels of leukotriene B_4_ and prostaglandin E_2_ *in vitro* in HIV-1 infected monocytes [46], and modulating the expression of genes in HIV-infected cells [88].

The anti-viral activity of avarone [47], a structurally similar compound also from the marine sponge *Disidea avara,* and its derivatives [48] has also been reported. Several new derivatives of avarol showing antiviral activities have also been extracted from the Red Sea sponge *Dysidea cinerea* [49]. The first enantioselective total synthesis of avarol was reported by Ling *et al.* [50]. In another attempt, the primmorph model (*in vitro* culture of sponge cells) was used as a model system to produce avarol in the laboratory [51]. The cell culture and gene cluster approaches used for sustainable production of avarol have also been reviewed [52].

### 2.3. Cyclic Depsipeptides

#### 2.3.1. Papuamide A, B, C, and D

**Figure 4:**
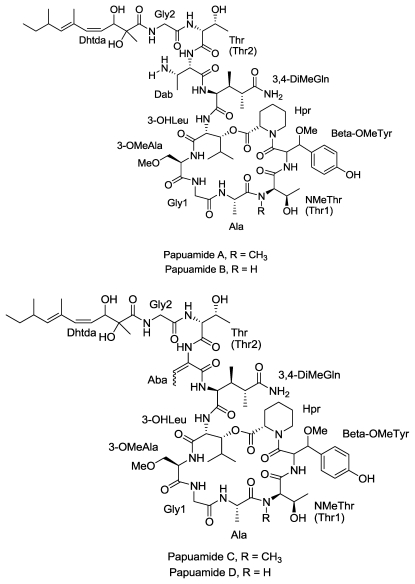
Structures of Papuamide A, B, C and D.

The anti-HIV and cytotoxic cyclic depsipeptides, papuamides, were isolated from the sponges *Theonella mirabilis* and *Theonella swinhoei* that were collected along the north coast of Papua New Guinea [53]. Two groups from the National Cancer Institute and the University of British Columbia independently reported the isolation of papuamides A and B from *T. mirabilis* and papuamides A, B, C, and D from *T. swinhoei*, respectively (Figure 4).

Extensive NMR analysis confirmed the presence of different amino acid residues, including alanine, threonine, two glycine residues, homoproline, *N*-methylthreonine, 3-methoxyalanine, β-methoxytyrosine, 3-hydroxyleucine, 3,4-dimethylglutamine, 2,3-diaminobutanoic acid and an amide linked 2,3 dihydroxy-2,6,8-trimethyldeca-(4*Z*,6*E*)-dienoic acid [54]. Papuamides A and B have been evaluated for their anti-HIV activity in cell based assays in CEM-SS T-cell cultures, and found to be highly potent with an effective concentration of3.6 ng/mL [54]. Activities for both compounds were found to be virtually identical.

Detailed mechanistic studies for the anti-HIV activity of papuamides A and B have been performed by Andjelic *et al.* [55]. Inhibition of viral entry into cells is shown to be independent of CD4, gp120, chemokine co-receptors and gp41, key proteins which are involved in the process of viral entry and are the targets of most of the FDA approved inhibitors of this process [56]. The mechanism of a direct interaction of papuamide A with the virus has been proposed witha membrane targeting mechanism believed to be responsible for the virucidal activity of the compound [55]. A similar type of mechanism has been proposed for an antifungal sterol dependent lipopeptide [57]. Papuamide B also inhibited viral entry at a concentration of 710 nM, with the proposed mechanism of targeting phosphatidylserine, a phospholipid present on the viral membrane. Papuamides C and D were found to be less potent with 30% and 55% inhibition at a concentration of 40 and 20 fold higher than papuamides A and B. In a recent study Xie *et al.* reported the total synthesis of papuamide B [54].

#### 2.3.2. Microspinosamide

**Figure 5:**
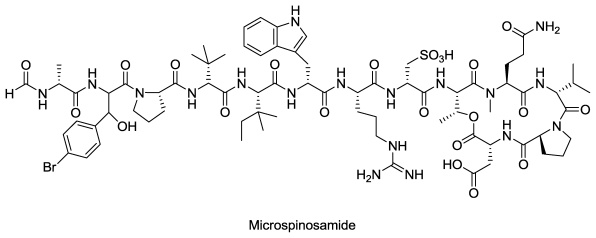
Structure of microspinosamide.

Isolation of microspinosamide, a cyclic depsipeptide (Figure 5), from an Indonesian collection of the sponge *Sidonops microspinosa* was reported in 2001 [58]. Microspinosamide contained 13 amino acid residues including alanine, tryptophan, arginine, threonine, aspartate, valine, two prolines, tert-leucine, β-methylisoleucine, *N*-methylglutamine, cysteic acid and a new residue, β-hydroxy-*p-*bromo-phenylalanine. The Anti-HIV activity of crude extract of *S. microspinosa* was first discovered during the National Cancer Institute’s primary anti-HIV screening [59]. Both aqueous and organic extracts of *S. microspinosa* exhibited anti-HIV activity. Microspinosamide was also evaluated for anti-HIV activity in a cell based in *vitro* assay and found to be effective at a concentration of 0.2 μg/mL in CEM-SS arget cells. Other cyclic depsipeptides from sponges with anti-HIV activity have also been reported [53,60,61].

### 2.4. Alkaloids

#### 2.4.1. 4-Methylaaptamine

**Figure 6:**
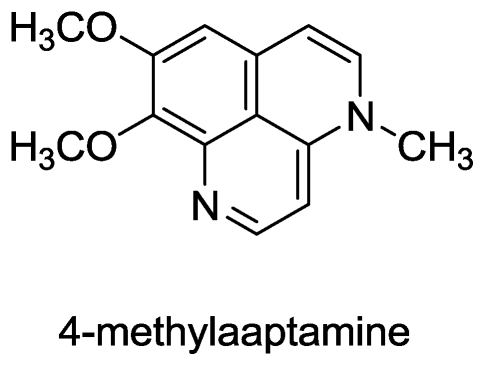
Structure of 4-methylaaptamine.

Isolation of the alkaloid 4-methylaaptamine (Figure 6) from the marine sponge *Aaptos sp.* (collected in Abrolhos, Bahia, Brazil) and the preliminary activity of its crude extract to inhibit 76% of HSV-1 replication in Vero cells at a concentration of 2.4 μg/mL was first reported by Coutinho *et al.* [62]. Another study confirmed the anti-HSV-1 activity of 4-methylaaptamine with an EC_50_ of 2.4 μM [63], which is even more potent than acyclovir, which has an EC_50_ of 8.6 μM [62]. 4-Methylaaptamine was found to inhibit HSV-1-infection in Vero cells even 4 h after infection, suggesting the inhibition of initial events during HSV-1 replication. Apparently the compound could inhibit expression of an HSV-1 immediate-early protein, ICP27, which regulates splicing, termination, and nuclear export of viral transcripts thus preventing viral replication [63].

Synthetic transformation of methylaaptamine, which was first isolated by Nakamura and co-workers [64], into 4-methylaaptamine has also been reported [65]. 9-*O*-4-Ethylbenzoylisoaaptamine, a novel derivative of isoaaptamine also displays potent activity against HIV-1 with an EC_50_ of 0.47 μg/mL [66].

#### 2.4.2. Dragmacidin F

**Figure 7:**
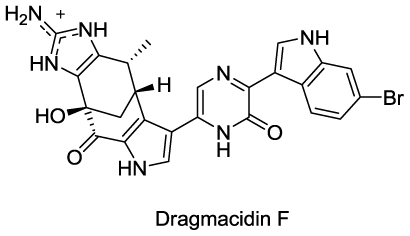
Structure of Dragmacidin F.

Cutignano *et al.* reported the isolation of a new bromoindole alkaloid, dragmacidin F (Figure 7), from a marine sponge of the genus *Halicortex* collected off the southern coast of Ustica Island (Italy) [67].

The compound demonstrated *in vitro* antiviral activity against HSV-1 and HIV-1 with an EC_50_ of 96 μM and EC_50_ of 0.9 μM respectively and hence is most likely responsible for the antiviral property exhibited by *Halicortex* extracts. The compound has an unprecedented carbon skeleton that is presumed to be derived biosynthetically from dragmacidin D by the cyclization of its partially oxidized form [67]. Total synthesis of (+)-dragmacidin F has been described by Garg *et al.* [68].

#### 2.4.3. Manzamine A

**Figure 8:**
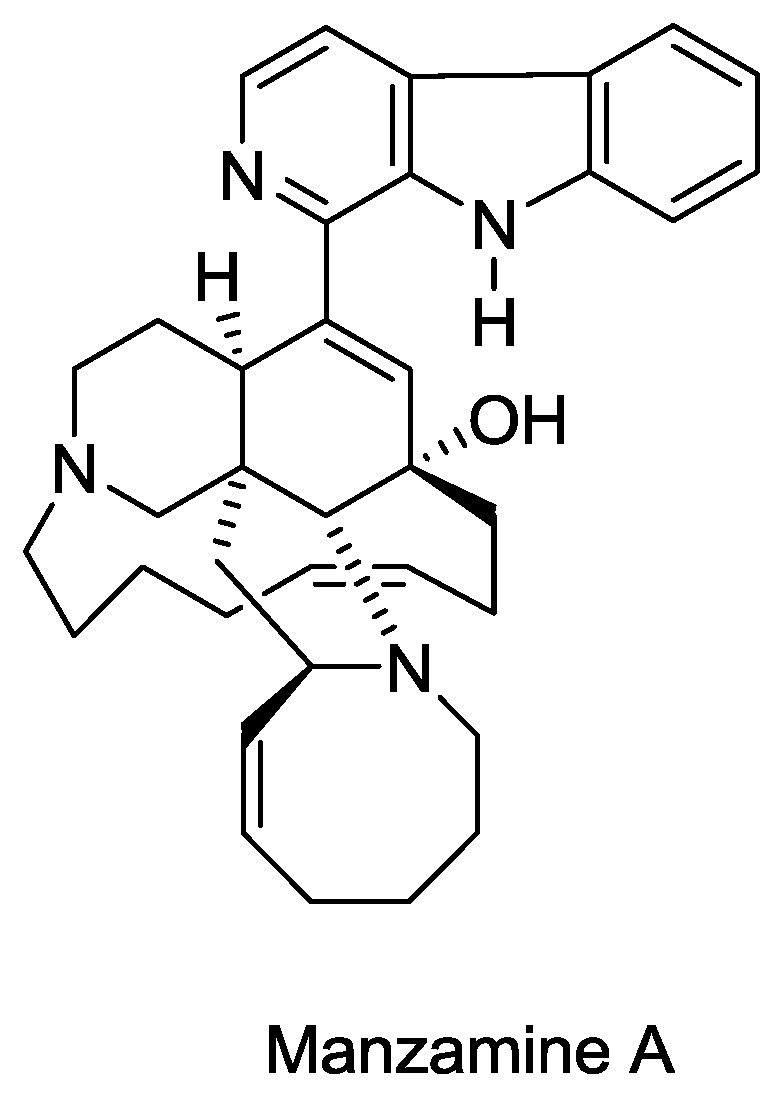
Structure of manzamine A.

Manzamine A (Figure 8) was isolated from *Haliclona sp.* Found in waters near Okinawa (Japan) by Sakai and Higa in their quest to find antitumor compounds from marine organisms [69]. The manzamine class of alkaloids has unique complex polycyclic ring systems coupled with a β-carboline moiety and has been reported to have a diverse range of bioactivities, including antimicrobial [70,71], antiparasitic [72], antipesticidal [73], and anti-HIV-1 and activity against AIDS opportunistic infections [74]. Isolation of manzamine A from the sponge *Pachypellina sp.* (Porifera, Demospongia, Petrosida, Oceanapiidae) collected at Manado Bay, Sulawesi, Indonesia has also been described [75]. The same study reported the first anti HSV-II activity of this compound with a minimal effective concentration of 0.05 μg/mL. Isolation of manzamine A has also been reported from other species of marine sponges [76–78]. Enantioselective total synthesis of manzamine A has been described by Humphrey *et al.* [79].

A more recent study describes the isolation of manzamine A from an undescribed sponge of the genus*Acanthostrongylophora* from Manado Bay, Indonesia; and its key oral and intravenous pharmacokinetic properties in rats have also been reported [80]. This study, which was the first published information regarding the pharmacokinetic properties of manzamine A, indicated that the compound has a low metabolic clearance, a reasonably long pharmacokinetic half-life, and good absolute oral bioavailability, making it a promising potential lead for further preclinical assessment and possible development. This study also reported the anti-HIV-1 activity of manzamine A, 8-hydroxymanzamine A, 6-deoxymanzamine X, and neokauluamine with EC_50_ of 4.2, 0.6, 1.6, and 2.3 μM, respectively.

### 2.5. Phenolic Macrolides

#### Hamigeran B

**Figure 9:**
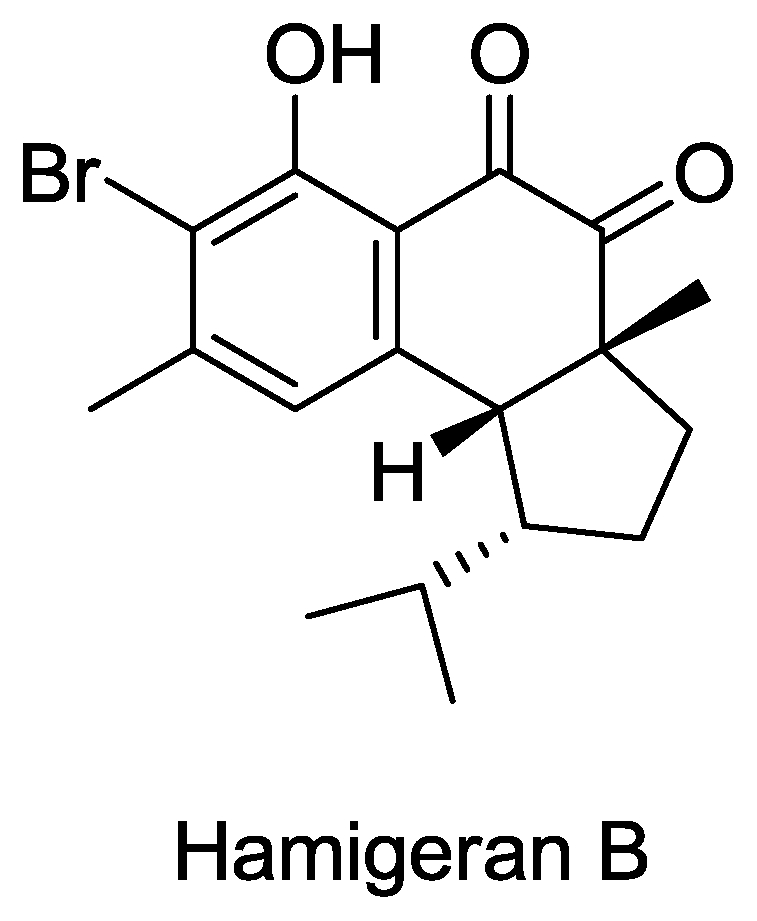
Structure of hamigeran B.

This compound (Figure 9) was isolated from the marine sponge *Hamigera tarangaensis* (family Anchinoidae) from the Hen and Chicken Islands in New Zealand and showed 100% *in vitro* virus inhibition against both the herpes and polio virus with only slight cytotoxicity at a concentration of 132 μg per disk [51]. Syntheses of hamigeran B have been reported by several groups 1 [81–83].

## 3. Discussion

A total of 40 compounds have been officially approved for clinical use in the treatment of various viral ailments and at least half of them are used for the treatment of HIV infection [84]. Most of the sponge-derived compounds have also been screened for anti-HIV activity, showing the interest and potential importance of this field. This has led to the discovery of many compounds with anti-HIV activity, such as avarol, microspinosamide, papuamides A–D *etc.* Although many antiviral lead compounds have been derived from sponges, none of them has yet been approved as a drug (except Ara-A which is no longer in use). One of the reasons for this is the difficulty in obtaining a sustainable supply of these complex molecules for pre-clinical and clinical trials [85]. Most of the pharmaceutically interesting compounds found in sponges are present in minute amounts. For example, in order to obtain even 300 mg of halichondrins, a potent cytostatic polyketide of sponge origin, 1 metric ton of the sponge *Lissodendoryx sp.* must be extracted [86]. In addition, it is difficult to chemically synthesize most of these compounds due to their highly complex structures. In addition, the very long drug development process [87] makes this problem even more challenging. It is clear that such a large amount of biomass of marine sponges cannot be harvested from nature, and in the event that it were it would put these species at risk of extinction. More environmentally friendly and economically feasible strategies are clearly needed. Mariculture of sponges for large scale production of these compounds is an option but insufficient knowledge of the conditions and specific parameters for the growth and cultivation of sponges in the laboratory are the limiting factors. Culturing cells and primmorphs for production of metabolites may be feasible in the future but at present this technique is unable to produce large amount of biomass [88].

A growing body of evidence suggests that marine natural products may be the products of bacterial symbionts of sponges [89,90]. The Faulkner group demonstrated for the first time that natural products from sponges could be of bacterial origin [91]. Microorganisms associated with sponges have been characterized into 14 different phyla and their diversity and biotechnological importance have been reviewed [92]. Isolation and cultivation of sponge-associated microorganisms (microbial fermentation) producing the bioactive natural products is also another option for the large scale production of compounds of interest [93,94]. The success of this strategy depends on many factors. The majority of sponge associated microorganisms are difficult to culture [95,96]. Improved culturing of sponge associated microorganisms by supplementing the media with sponge extract [97] or catalase and sodium pyruvate [98] has been reported, but the proportion of total cultured bacteria has remained low. Only 0.06 and 0.1% of total bacteria could be cultured from the sponges *Candidaspongia flabellate* [99] and *Rhopaloeides odorabile* [97]. Furthermore, microorganisms isolated from sponges may not necessarily produce the same compound due to the requirement of intermediate compound/s from the host. Some bacteria also stop producing the compound of interest after a certain time on artificial media, which may be caused by a number of genetic factors linked to lack of selective pressure in culture [100]. To develop successful sponge culturing methods it is essential to understand the biology and natural living conditions of the sponges affecting growth and metabolite production. Various methods to culture sponges and sponge symbionts have been reviewed previously [101,102]. The attempts to develop and grow *in vitro* cell lines from sponges from metabolite production have also been reported [103].

Metagenomics is another strategy that has been used successfully to identify the biosynthetic origin of natural products. This procedure involves the genomic analysis of the total DNA in an organism and its symbionts. In the past few years metagenomics has emerged as a potential solution for genetic characterization of unculturable bacteria associated with marine sponges [104]. The method involves direct extraction and cloning of DNA from a group of bacteria and its genomic sequencing [105]. Initial efforts included the identification and isolation of gene clusters responsible for production of secondary metabolites involved in biosynthetic pathways, such as polyketide synthase (PKS) gene clusters [106,107]. Another study reported the cloning of chondramide biosynthesis cluster from *C. crocatus, a* myxobacterium [108]. The metagenomic approach was also employed for characterizing sponge-specific candidate phylum “*Poribacteria*” [109,110] and a new molybdenum-containing oxidoreductase and transmembrane proteins were identified [110]. The gene clusters identified using metagenomics approach is a step forward towards solving the problem of mass production of relevant natural products which further depends on the expression of the isolated gene clusters in relevant host. Heterologous expression vectors have been used to express the PKS biosynthetic clusters in *Pseudomonas putida* [111,112]. Other examples of expression hosts include *E. coli* [111–115], *Myxobacteria* and *Streptomyces* [116,117] used for expression of various biosynthetic pathways. Long *et al.* [118] applied the expression based techniques to identify expressing clones. The isolation of compounds from marine metagenomes is successful to a limited extent but this technology has been effectively employed on soil metagenomes where several antibiotics have been isolated using metagenomic approaches [119–122]. Although these studies demonstrate the success achieved by using the metagenomics approach there are still some technological issues related to this approach which must be overcome. Studies have provided compelling evidences that natural products known as polyketides are structurally similar in sponges and symbiont bacteria [2,123]. It has been made clear that these bacteria are the key producers of polyketides [124,125]. The complexity of the genomes of the group of organisms makes it very difficult to identify the target genome, and is further complicated by the use of inappropriate host organisms for cloning and expression [105,126] as well as the large size of the gene clusters [127]. The obstacles are manifold since sponges play host to a wide diversity of organisms such as bacteria, fungi, protists etc [128] resulting in a complex community. The expression of such complex metagenome will not be feasible in simple expression systems such as *E. coli*. The complex expression systems are needed to achieve the success in case of sponges [104]. To overcome the challenges associated with successful implementation of metagenomics approach, new methods have been developed and tested recently. One possible future direction could be to perform sequence based screens in order to identify enzymes that have been shown to be involved in the synthesis of anti-viral compounds. This strategy has been successfully developed and implicated to known polyketide synthase genes in an effort to identify new polyketides [129]. Other recently developed phylogenetic approaches can be applied to study the structure and function of biosynthetic enzymes as well as to isolate target gene clusters [130]. The metagenomic libraries can also be screened for antiviral activities by tailoring the methodologies previously used to identify natural-product clusters using genome sequence tags (GSTs). GSTs are the parts of the genes that can be used as probes to screen for similar genes in a clonal library. Any clone containing a GST can be a potential candidate for screening of novel natural-product gene clusters. This approach has been utilized to identify more than 450 natural-product clusters [131].

## 4. Conclusions

The literature regarding antiviral compounds from sponges shows the significance of marine natural products in the drug discovery and development process. With advancement of technologies a new generation of potent and effective antiviral agents may be obtained from these sources. Sequence based screens, metagenomic clonal library screening using GSTs and other phylogenetic approaches could provide a new future dimension in search for antiviral natural compounds from sponges. The successes in metagenomics coupled with heterologous expression and high throughput microbial cultivation techniques could pave the way for commercial production of such compounds in the future, greatly facilitating their analysis and commercialization.
